# Microglia activation linking amyloid-β drive tau spatial propagation in Alzheimer's disease

**DOI:** 10.3389/fnins.2022.951128

**Published:** 2022-08-12

**Authors:** Qing Wang, Chunming Xie

**Affiliations:** ^1^Department of Neurology, Affiliated ZhongDa Hospital, School of Medicine, Southeast University, Nanjing, China; ^2^Institute of Neuropsychiatry, Affiliated ZhongDa Hospital, Southeast University, Nanjing, China; ^3^The Key Laboratory of Developmental Genes and Human Disease, Southeast University, Nanjing, China

**Keywords:** neuroinflammation, Alzheimer's disease, microglial activation, tau pathology, PET imaging

Alzheimer's disease (AD) is the most common cause of dementia, accounting for an estimated 60–80% of diagnosed cases, and affecting 33.2% of individuals older than 85 years (Alzheimer's Association, 2022). AD is characterized by a progressive cognitive decline, with impairment in multiple cognitive domains, including memory, executive function, and language (Mckhann et al., 2011). The pathological hallmarks of AD are extracellular amyloid-β (Aβ) plaques and intraneuronal neurofibrillary tangles (NFTs) of tau protein (Braak and Braak, 1991). Following the discovery of elevated levels of inflammatory markers for patients with AD as well as of AD-risk genes associated with innate immune functions, neuroinflammation mediated by microglia has emerged as a vital player (Heppner et al., 2015; Calsolaro and Edison, 2016).

Microglia is the resident innate immune cells of the central nervous system that is capable of disease progressions by altering their physiological function and inducing the activation of inflammatory pathways (Shemer et al., 2015). The presence of toxic Aβ and tau proteins is believed to activate microglia. Microglia tries to clear the toxic proteins as well as widespread debris from dead and dying cells (Marín-Teva et al., 2004; Kettenmann et al., 2011). Chronic inflammation may set in when microglia cannot keep up with all that needs to be cleared, resulting in neuronal dysfunction, injury, and loss (Streit et al., 2004; Feng et al., 2014). In addition, other external factors may affect the pro-inflammatory function of microglia (i.e., reactive astrocyte, BBB leaking) (Streit et al., 2004; Chen et al., 2017; Zhao et al., 2020). Recent findings using positron emission tomography (PET) scanning have clearly shown that microglia activation and neuroinflammation are highly correlated with cognitive decline in patients with AD (Hamelin et al., 2016; Terada et al., 2019). Autopsy of patients with AD revealed both extracellular Aβ plaques and neurons containing NFTs are surrounded by activated microglia, consistent with animal experiments reports (Serrano-Pozo et al., 2011; Li et al., 2013; Sanchez-Varo et al., 2021).

Using voxel-based biological parametric mapping, previous PET studies have also confirmed that microglial activation is strongly correlated with both tau tangle load and amyloid deposition in subjects with mild cognitive impairment (MCI) and AD (Dani et al., 2018; Ismail et al., 2020). Additionally, abnormal tau deposition was observed in temporo-parietal cortices, like that of microglia activation in both the MCI (amyloid-positive and amyloid-negative) and AD subjects (Dani et al., 2018; Terada et al., 2019). Furthermore, microglia activation in cell and murine models exacerbates tau hyperphosphorylation, aggregation, and neurodegeneration (Maphis et al., 2015; Clayton et al., 2021). Tau pathology induces microglia activation, suggesting a vicious cycle between tau pathology and activated microglia (Morales et al., 2013; Maphis et al., 2015). However, it remains poorly understood how microglial activation drives tau tangles spatially propagation in patients with AD pathophysiology.

Fortunately, in an elegant study, using PET imaging for microglial activation [(11C) PBR28], β-amyloid (Aβ) [(18F) AZD4694], and tau [(18F) MK-6240], Pascoal et al. (2021) *in vivo* demonstrated the relationships of these three key pathological features in 130 individuals across the spectrum of aging and AD. With the use of both cross-sectional and longitudinal analyses, they found that microglial activation and tau accumulation spatially propagate in parallel, following brain circuits predicted by postmortem series from the transentorhinal/entorhinal to sensorimotor cortices. Moreover, their findings support that Aβ potentiates the effects of microglial activation on tau spreading. Furthermore, they reported that the co-occurrence of Aβ, tau pathology, and microglia abnormalities was synergistically associated with the development of cognitive impairment and dementia. These lines of evidence suggest that future therapies that target the immune neuroinflammation system may slow the spread of tau in patients with early AD. The complex interactions between the three hallmarks of AD have become an intriguing topic of study.

Microglial activation driving tau pathology is contingent on the microglial circuits. Tau pathology is assumed to propagate along a network of interrelated brain regions, following an anatomical sequence first defined by Braak and Braak (1997). Pascoal et al. demonstrated that microglial activation follows a similar pattern in AD and called it “microglial circuits”. One study found microglia produces and secretes extracellular vesicles and accelerates tau propagation in a humanized APP mouse model (Clayton et al., 2021). Another study found microglia contains and releases tau seeds in a transgenic mouse model for AD (rTg4510) as well as in brain samples from patients with human AD, suggesting that they play a direct role in propagation of tau pathology throughout the brain (Hopp et al., 2018). Moreover, recent studies on animal models have suggested that microglial activation drives tau pathology *via* activating NLRP3 inflammasome or regulating microglial fractalkine receptor (Bhaskar et al., 2010; Ising et al., 2019). Thus, it takes a very important step toward a comprehensive and in-depth understanding of how microglial activation affects AD pathology and may be incorporated in the ATN framework underlying an AD mechanism in the future.

Why microglial activation could influence the distribution of tau? In the pathological progression of AD, different species of Aβ aggregates, tau oligomers and fibrils, and damaged neurons could induce microglia activation and the production of pro-inflammatory cytokines and chemokines, all of which could lead to neuronal dysfunction and death (Sondag et al., 2009; Heppner et al., 2015; Hansen et al., 2018). Usually, microglia promotes the release of inflammatory factors by activating the NLRP3 inflammasome (Ising et al., 2019). Early studies established that Aβ can act as a danger-associated molecular pattern to activate pattern recognition receptors on the microglia surface, such as Toll-like receptors, and induce activation, secretion, and phagocytosis of microglia cells (Venegas and Heneka, 2017; Alawieyah Syed Mortadza et al., 2018). Aβ can also directly interact with amyloid precursor protein in a concentration-dependent manner to jointly induce microglia activation and secretion of inflammatory factors (Manocha et al., 2016). Further experiments have found that pathogenic Aβ and tau pathology might contribute to the dystrophy of microglia, which can accelerate neuronal loss, leading to more deposition of NFT and amyloid beta *in vitro and in vivo* (Nussbaum et al., 2013). Moreover, *in vitro* studies have found that tau oligomers and fibrils provide a sufficient stimulus to induce microglial morphological change and expression of interleukins (Morales et al., 2013). In addition, previous studies in animals have suggested that microglial activation exacerbates tau pathology, at least partially *via* tau kinases in an IL-1β-dependent manner (Ising et al., 2019) and the neuronal IL-1R–p38 MAPK pathway (Bhaskar et al., 2010). There is another study that shows Aβ plaques promoting the spread of tau in mice (Bassil et al., 2020). Other existing reports on transgenic animals and human AD brain samples have highlighted microglia as a potential bridge between Aβ plaques and tau (Kitazawa et al., 2004). Consequently, the relationship between Aβ burden, tau, and microglial activation remains unclear. With statistical modeling, this study provides the strongest evidence that Aβ deposition has a synergistic interaction with microglial activation on the topographical spread of tau pathology. However, it is not clear whether Aβ is involved in mediating the effect of microglial activation on tau pathology or whether microglial activation mediates the effect of Aβ on tau pathology.

The most interesting finding is that only the concomitant presence of Aβ, tau, and microglial activation abnormalities could elicit the cognitive impairment. This is consistent with the previous findings that AD pathology accumulates early before clinical symptoms (Jack et al., 2013). These data suggest, on the one hand, that early pathological changes in AD spectrum may not affect cognitive function. On the other hand, the limitation of MMSE that could not detect subtle cognitive dysfunction needs to be acknowledged. Therefore, future clinical studies should employ a higher sensitivity toolkit of cognition detection and assessment.

Nevertheless, some essential issues still should be further elucidated.

First, the current results demonstrated that microglial activation facilitates transmission of tau pathology. However, factors are involved in the process when microglial activation spurs tau aggregation is still a mystery.

Second, [11C] PBR28 is a second-generation PET radioligand with high specific binding to the 18-kDa translocator protein (TSPO), which is expressed by microglia as well as reactive astrocytes. Evidence of animal studies has indicated that increased TSPO density could reflect acute injury with associated activation of microglia, or, alternatively, could represent past damage with residual astrocytosis (Lavisse et al., 2012; Librizzi et al., 2012). Increased [11C] PBR28 binding in AD, therefore, does not necessarily reflect ongoing activation of microglia. However, early results from transgenic mouse and autopsy studies have shown that the increased TSPO density in the AD brain co-localizes to microglia rather than astrocytes (Venneti et al., 2008; Gulyas et al., 2009). Beyond that, microglial activation is a complex process that produces multiple phenotypes (Prater et al., 2021; Wang, 2021), while TSPO PET tracers cannot robustly distinguish between these distinct phenotypes. Thus, caution must be used when interpreting TSPO PET imaging in animals or patients.

Third, microglial activation is highly heterogeneous, not only between disease types but also different patients with the same disease. Microglial activation occurs in nearly all neurodegenerative diseases (Perry et al., 2010; Shemer et al., 2015; Tang and Le, 2016; Salter and Stevens, 2017), and likely involves multiple mechanisms. Aggregated α-synuclein is a major component in intraneuronal inclusions known as Lewy bodies associated with Parkinson's disease and dementia with Lewy body. Experimental studies using knockout mice have shown that microglia play a neuroprotective role by clearing α-synuclein through tlR4-NF-κB-P62-mediated eukaryotic phagocytosis (Choi et al., 2020). Additionally, an *in vivo* PET study showed that microglia activation correlates with severity of the pathology in patients with Huntington's disease (Crotti et al., 2014). Furthermore, small vessel disease can be associated with microglial activation, which is a well-recognized subacute response to ischemic stroke (Venneti et al., 2008; Maida et al., 2020). Thus, the presence of microglial activation may be related to various physiologic and pathologic processes, with tau hyperphosphorylation representing the end of a final common pathway.

Recent studies have shown that there are two peaks of microglial activation in the Alzheimer's disease trajectory *in vivo*: an early protective peak and a later pro-inflammatory peak (Hamelin et al., 2016; Fan et al., 2017; Ismail et al., 2020). A mouse study crossing transgenic amyloid and transgenic tau mice produced offspring with increased microglial activation (and increased phagocytic ability), and a 40–50% reduction in Aβ plaque load and insoluble Aβ species, implying that, under certain circumstances, tau-induced microglia activation clears amyloid load (Chen et al., 2016). An *in vivo* PET study suggests microglial activation appears at the prodromal and, possibly, at the preclinical stage of AD, and seems to play a protective role in the clinical progression of the disease at these early stages (Hamelin et al., 2016; Fan et al., 2017). If so, immunotherapy would be a reasonable approach for achieving this in early disease stages. However, current PET tracers are unable to differentiate between protective or detrimental roles of activated microglia. In addition, the distributions of the TSPO PET tracer were different from nine patients with AD, emphasizing the heterogeneity of pathologies in individuals (Dani et al., 2018). For all, microglial activation represented lower sensitivity and higher specificity in different populations. Future studies are warranted to further explore metrics for significant heterogeneity of microglial activation between individuals, diseases, or disease states.

Overall, although many issues remain open, Pascoal et al. firstly revealed that microglial activation sets the stage for the stereotyped spread of tau pathology in Braak stages in patients with AD. Furthermore, they found that Aβ potentiates the effects of microglial activation on tau spreading. More importantly, co-occurrence of Aβ, tau, and microglia abnormalities seems necessary for a full-bloom dementia phenotype. Particularly, the results may have implications for clinical trials: (1) individuals with tau deposition confined to medial temporal regions would benefit from preventive therapies targeting on neuroinflammation. (2) These clinical trials could have advantages from using well-validated fluid or brain imaging markers of microglial activation to assess drugs target engagement and efficacy. These results suggest that preventative treatment for AD should target on all three processes.

## Author contributions

QW drafted the original manuscript. CMX critically revised the manuscript. All authors agreed to be accountable for the content of the work. Both authors contributed to the article and approved the submitted version.

## Funding

This work was supported by the Science and Technology Innovation 2030 Major Projects (2022ZD0211600), National Natural Science Foundation of China (81671256, 81871069, and 82071204), and the Key Projects of Jiangsu Commission of Health (ZDB2020008).

## Conflict of interest

The authors declare that the research was conducted in the absence of any commercial or financial relationships that could be construed as a potential conflict of interest.

## Publisher's note

All claims expressed in this article are solely those of the authors and do not necessarily represent those of their affiliated organizations, or those of the publisher, the editors and the reviewers. Any product that may be evaluated in this article, or claim that may be made by its manufacturer, is not guaranteed or endorsed by the publisher.
